# Bag of Naïve Bayes: biomarker selection and classification from genome-wide SNP data

**DOI:** 10.1186/1471-2105-13-S14-S2

**Published:** 2012-09-07

**Authors:** Francesco Sambo, Emanuele Trifoglio, Barbara Di Camillo, Gianna M Toffolo, Claudio Cobelli

**Affiliations:** 1 Department of Information Engineering, University of Padova, 35131 Padova, Italy

## Abstract

**Background:**

Multifactorial diseases arise from complex patterns of interaction between a set of genetic traits and the environment. To fully capture the genetic biomarkers that jointly explain the heritability component of a disease, thus, all SNPs from a genome-wide association study should be analyzed simultaneously.

**Results:**

In this paper, we present Bag of Naïve Bayes (BoNB), an algorithm for genetic biomarker selection and subjects classification from the simultaneous analysis of genome-wide SNP data. BoNB is based on the Naïve Bayes classification framework, enriched by three main features: bootstrap aggregating of an ensemble of Naïve Bayes classifiers, a novel strategy for ranking and selecting the attributes used by each classifier in the ensemble and a permutation-based procedure for selecting significant biomarkers, based on their marginal utility in the classification process. BoNB is tested on the Wellcome Trust Case-Control study on Type 1 Diabetes and its performance is compared with the ones of both a standard Naïve Bayes algorithm and HyperLASSO, a penalized logistic regression algorithm from the state-of-the-art in simultaneous genome-wide data analysis.

**Conclusions:**

The significantly higher classification accuracy obtained by BoNB, together with the significance of the biomarkers identified from the Type 1 Diabetes dataset, prove the effectiveness of BoNB as an algorithm for both classification and biomarker selection from genome-wide SNP data.

**Availability:**

Source code of the BoNB algorithm is released under the GNU General Public Licence and is available at http://www.dei.unipd.it/~sambofra/bonb.html.

## Background

In the past few years, the hereditary component of complex multifactorial diseases has started to be explored through the novel paradigm of Genome-Wide Association Studies (GWASs). A GWAS searches for patterns of genetic variation, in the form of Single Nucleotide Polymorphisms (SNPs), between a population of affected individuals (cases) and a healthy population (controls). The objective of a GWAS is twofold: on the one hand, one searches for the set of SNPs that best explains the hereditary component of the disease (*genetic biomarkers*); on the other hand, one tries to learn a rule for classifying unknown subjects as cases or controls, given their genetic profile and possibly other environmental covariates [1].

Further applications of GWASs include searching for the genetic predisposition to complex traits, such as height [2] or Body Mass Index [3], or to the responsiveness to a treatment in a randomized trial [4]. The application of GWAS is not limited to human: successful results have been obtained by applying the GWAS framework to animal [5] and plant research [6].

The extremely large numbers involved in a GWAS (millions of SNPs measured for thousands of individuals) have led the vast majority of studies to rely upon single, univariate SNP association tests [7-9]. Multifactorial diseases, however, have an heterogeneous nature, arising from complex patterns of interaction between a set of genetic traits and the environment: to fully capture the optimal set of genetic biomarkers, thus, all SNPs in a GWAS should be analyzed simultaneously in a multivariate framework [10].

In the literature, the few approaches to multivariate SNP analysis on a genome-wide scale mainly rely on two methodological frameworks: penalized logistic regression [10-12] and Bayesian analysis [13]. In the first case, SNPs are modelled as discrete variables from the finite domain {0,1,2} (where 0 usually encodes the homozygous pair of minor alleles, 1 the heterozygous pair and 2 the homozygous pair of major alleles) and a log-additive model of genetic effect on the disease is assumed. In the second case, SNPs are modelled as ternary categorical variables and no assumptions are usually made on pre-specified genetic models.

All methods for the simultaneous analysis of the whole SNP set have to cope with *genetic linkage*, *i.e*. the non random association between portions of the genome close to each other, which acts as a confounding factor: in the proximity of a true causal genetic biomarker, several SNPs highly correlated with the biomarker but mildly associated to the disease are often observed [14].

In this work, we present Bag of Naïve Bayes (BoNB), an algorithm for classification and genetic biomarker selection from the simultaneous analysis of genome-wide SNP data. Our algorithm is based on Naïve Bayes (NB) classification [15], thus it relies on contingency table analysis and it does not assume a pre-specified model of genetic effect.

Three strategies are exploited in BoNB to tailor the Naïve Bayes framework to genome-wide SNP data analysis: (a) a bagging of Naïve Bayes classifiers, to improve the robustness of the predictions, (b) a novel strategy for ranking and selecting the attributes used by each bagged classifier, to enforce attribute independence, and (c) a permutation-based procedure for selecting significant biomarkers, based on their marginal utility in the classification process.

BoNB is tested on the WTCCC case-control study on Type 1 Diabetes [7]. In terms of classification performance, assessed through repeated random sub-sampling cross validation and measured with the Matthews Correlation Coefficient [16], BoNB outperforms both a standard Naïve Bayes classifier, trained on the SNPs that reached genome-wide significance in a univariate test, and HyperLASSO, a state-of-the-art penalized logistic regression technique specifically designed for the simultaneous analysis of genome-wide data [10].

## Results

### Algorithm

Given a dataset **X**, consisting of *n *observations (subjects) of *p *attributes (SNPs), and a set **Y **of class labels, one for each observation (case/control), a Naïve Bayes Classifier (NBC, [15]) estimates from the dataset a classification rule in the form:

(1) $$\text{Pr}\left(Y=y_{k}|X_{1}\ldots X_{p}\right)=\frac{\text{Pr}\left(Y=y_{k}\right)\prod_{i}\text{Pr}\left(X_{i}|Y=y_{k}\right)}{\sum_{j}\text{Pr}\left(Y=y_{j}\right)\prod_{i}\text{Pr}\left(X_{i}|Y=y_{j}\right)},$$

where *Y *is the random variable representing the class label and *X*_1 _. . . *X_p _*are the random variables representing the *p *attributes.

The classification rule of Equation (1) states that the probability of a subject being in class *y_k_*, given a combination of values for the attributes *X*_1 _. . . *X_p_*, is equal to the *a priori *probability of class *y_k_*, Pr(*Y *= *y_k_*), times the probability of each attribute given class *y_k_*, Pr(*X_i_*|*Y *= *y_k_*): the implicit assumption below this classification rule is that attributes *X*_1 _. . . *X_p _*are all *conditionally independent *given *Y*.

For categorical attributes, such as SNPs, probability distributions Pr(*Y = y_k_*) and Pr(*X_i_*|*Y = y_k_*) are represented with conditional probability tables, which are estimated from the data by counting the occurrences of each combination of genotypes and class labels (see Methods for more details).

Our algorithm, Bag of Naïve Bayes (BoNB), consists in an ensemble of Naïve Bayes Classifiers, trained on GWAS data with the procedure known as Bootstrap Aggregating or *Bagging *[17].

**Figure 1:**
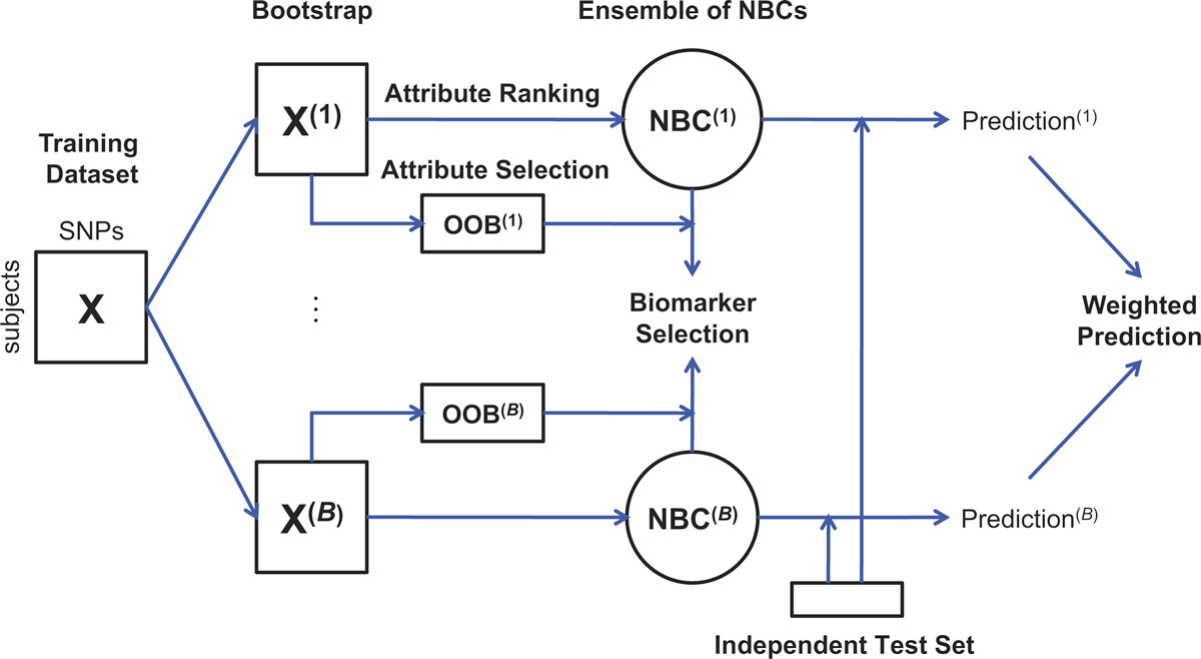
**Schematics of the BoNB algorithm**: *B *Bootstrap samples {**X**^(1) ^. . . **X**^(*B*)^} are drawn from a GWAS training dataset **X**; *B *Naïve Bayes Classifiers (NBC) are trained on the Bootstrap samples, with the novel procedure for attribute ranking and selection; predictions of unseen subjects from a GWAS test dataset are carried out independently by each NBC and class probabilities are then averaged; biomarker selection is carried out with the novel permutation-based procedure, exploiting Out-of-Bag (OOB) samples.

Given a training dataset **X**, the Bagging procedure starts by computing a set of *B Bootstrap replicates *of **X**, *i.e*. a set {**X**^(1) ^. . . **X**^(*B*)^} of datasets, each one obtained by sampling *n *observations with replacement from the training set **X **[18]. A Naïve Bayes Classifier NBC^(*b*) ^is then trained on each Bootstrap sample **X**^(*b*)^. Class probabilities of unseen subjects, drawn from an independent test set, are then obtained by averaging the output class probabilities computed by each NBC^(*b*) ^(Figure 1). Such an approach is known to increase the robustness of the predictions [17].

Given the binary nature of the case/control classification problem and the frequent unbalance between the number of cases and controls in a GWAS, we decided to rely on the Matthews Correlation Coefficient (MCC, [16]) for assessing classification performance. The MCC is defined as:

(2) $$MCC=\frac{tp\;\cdot \;tn-fp\;\cdot fn}{\sqrt{\left(tp+fp\right)\cdot \left(tp+fn\right)\cdot \left(tn+fp\right)\cdot \left(tn+fn\right)}},$$

where *tp*, *tn*, *fp *and *fn *stand for true positives, true negatives, false positives and false negatives, respectively.

The MCC is often preferred to standard classification accuracy, *i.e*. to the proportion of correctly classified examples, because it is not sensitive to class unbalance: the MCC, in fact, ranges from -1 (all examples incorrectly classified) to 1 (all correctly classified) and equals 0 in case of majority classification, *i.e*. when all labels are assigned to the most represented class.

The conditional independence assumption below the Naïve Bayes classification rule (Equation (1)) is unlikely to hold if all the SNPs of a GWAS are exploited as attributes, because of genetic linkage. Moreover, computing Equation (1) for the whole SNP set can be computationally cumbersome and can lead to numerical and overfitting problems.

We thus developed a procedure for selecting a good set of independent SNPs for each NBC^(*b*)^: the procedure consists of a ranking step followed by an attribute selection step. In the ranking step, each SNP is given a score, according to its ability in discriminating the subjects in the bootstrap sample **X**^(*b*)^. The score is thus defined as the MCC of a Naïve Bayes Classifier, trained and tested on the same set **X**^(*b*)^, with the SNP as a single attribute (a precise mathematical description of the Naïve Bayes attribute score is given in Methods). SNPs are then ranked in decreasing order of score.

In the attribute selection step, SNPs are iteratively extracted from the top of the ranked list and added as attributes of NBC^(*b*)^. Each time a SNP is included as an attribute, the procedure removes from the ranked list all the SNPs that are both close to the SNP on the genome (distance < 1 Mb) and correlated with it (*r*^2 ^>*θ*, where *r*^2 ^is the squared correlation between the two SNPs and *θ *is a user defined threshold, default = 0.1). Such an approach enforces attribute independence, thus coping with the problems arising from genetic linkage.

Rather than including one SNP at a time, uncorrelated SNPs are added in groups of exponentially increasing size, starting from one SNP and doubling the size at each new addition. New SNPs are added as long as the generalization ability of NBC^(*b*) ^increases: to estimate the generalization ability, we test each NBC^(*b*) ^on the corresponding Out-of-Bag sample OOB^(*b*)^, consisting of all the observations left out from **X **when sampling **X**^(*b*)^, and measure the MCC of the prediction. The exponential increase in the number of added attributes allows BoNB to reach the adequate size for the attribute set of each NBC in a logarithmic number of steps.

The attribute selection procedure, iterated for the *B *bootstrap samples, results in an ensemble of *B *Naïve Bayes Classifiers, each with a possibly different set of attributes. Classification of unseen subjects, the first objective of GWASs, is then obtained by averaging the output class probabilities across all NBCs. Classification performance of the ensemble of NBCs can then be assessed on an independent GWAS test set, by measuring the MCC of the predictions.

For the second objective of GWASs, biomarker selection, we adapted for BoNB a procedure originally designed for the Random Forests bagged classifier [19]: for each of the SNPs included as attributes by at least 5% of the NBCs, we randomly permute the genotype of the SNP in the OOB sets, test each NBC^(*b*) ^on its corresponding OOB^(*b*) ^and record the relative decrease in MCC due to the permutation. Such a measure, which we define *marginal utility *(MU), can be used as an indicator of the importance of each selected attribute, given all other selected attributes.

For each SNP, the permutation procedure returns a list of values of MU, one value for each NBC that included the SNP: we test for MUs significantly greater than zero with a one-tailed Wilcoxon signed rank test, selecting as biomarkers the SNPs for which the p-value of the test is lower than 0.05.

The following pseudocode summarizes the training phase and the biomarker selection phase of the BoNB algorithm:

BoNB(**X**, *Y*, *B*, *θ*)

// Training

1 **for ***b *= 1 **to ***B*

2    [**X**^(*b*)^, OOB^(*b*)^] = bootstrap replicate from **X**

3    **for ***s ***= **1 to *p*

4       Compute the contingency table for SNP *s *from **X**^(*b*)^

5       Compute the Naïve Bayes attribute score of *s*

6    *L*^(*b*) ^= list of SNPs in decreasing order of score

7    Initialize NBC^(*b*) ^as a Naïve Bayes Classifier with no attributes

8    Extract *M *= 1 new attributes for NBC^(*b*) ^from the top of *L*^(*b*)^, excluding from future additions all SNPs at distance > 1 Mb and with *r*^2 ^<*θ*

9    **while **MCC of NBC^(*b*)^, tested on OOB^(*b*) ^with the new attributes, increases

10       Add the new attributes to NBC^(*b*)^

11       Update *M *= 2 * *M*

12       Extract *M *new attributes from the top of *L*, excluding each time from future additions all SNPs at distance > 1 Mb and with *r*^2 ^<*θ*

// Biomarker selection

13 **for ***s ***in **all SNPs selected by at least 5% of the NBCs

14    **for ***b ***in **all NBCs that selected *s*

15       Permute the genotype of *s *in OOB^(*b*)^

16       Record the Marginal Utility (MU) of *s*

17 Select as biomarkers the SNPs with MU significantly larger than zero.

For analyzing the computational complexity of BoNB, one can start by noting that, for each *b *in *B*, the attribute ranking step (lines 3-6) has complexity *O*(*np*) for computing the contingency tables and the scores (where *n *is the number of subjects and *p *is the number of SNPs in the dataset) plus *O*(*p *log *p*) for sorting the score list, thus has a total complexity of *O*[*Bpn *+ *Bp *log *p*)].

The attribute selection step (lines 7-12), executed for each *b *in *B*, has a computational complexity dominated by two operations: computation of the squared correlation coefficient *r*^2 ^between SNPs and test of NBC^(*b*) ^on OOB^(*b*)^. If we define $\bar{M}$ the average number of attributes included by each NBC (which is problem dependent) and ${\bar{p}}_{1Mb}$the average number of SNPs in a 1 Mb section of the DNA (which is dataset dependent, but is a roughly linear function of *p*), we can note that the first operation costs *O*(*n*) for each SNP pair and is executed $\bar{M}\;\cdot \;{\bar{p}}_{1Mb}$ times, having thus a total computational complexity of $O\left(Bn\bar{M}\;{\bar{p}}_{1Mb}\right)$. The second operation, on the other hand, is executed log $\bar{M}+2$ times, each time with a doubling number of features for NBC^(*b*)^, and its computational complexity is thus expressed by the following summation:

$$\overset{\text{log }\bar{M}+1}{\underset{i=0}{\sum }}{\bar{n}}_{OOB}\;\cdot \;\;2^{i}={\bar{n}}_{OOB}\left(2^{\text{log }\bar{M}+2}-1\right)≅O\left(n\bar{M}\right),$$

where ${\bar{n}}_{OOB}$ is the average number of subjects in an OOB set, tending to (1 - 1/*e*) · *n *for large *n *[18]; the total complexity of the second operation is thus $O\left(Bn\bar{M}\right)$, asymptotically negligible with respect to the cost of computing the squared correlation coefficients. The total computational complexity of the training phase of the BoNB algorithm is thus $O\left[B\left(pn+p\text{ log }p+n\bar{M}{\bar{p}}_{1Mb}\right)\right]$.

For the complexity of the biomarker selection phase of BoNB, we define ${\bar{p}}_{\text{5}\%}$ the number of SNPs selected by at least 5% of NBCs (which is problem dependent) and note that the inner loop of lines 15-16 is executed at most $O\left(B{\bar{p}}_{\text{5}\%}\right)$ times; since the cost of the two operations in the loop is linear in *n*, the biomarker selection phase has a total computational complexity of $O\left(Bn{\bar{p}}_{5\%}\right)$.

### Testing

BoNB was tested on the WTCCC case-control study on Type 1 Diabetes [7]: the study examined approximately 2000 T1D cases and 3000 healthy controls. Each subject was genotyped on the Affymetrix GeneChip 500K Mapping Array Set.

We excluded a small number of subjects according to the sample exclusion lists provided by the WTCCC. In addition, we excluded a SNP if (i) it is on the SNP exclusion list provided by the WTCCC; (ii) it has a poor cluster plot as defined by the WTCCC. The resulting dataset consists of 458376 SNPs, measured for 1963 cases and 2938 controls.

The number *B *of Bootstrap replicates used by BoNB was set to 200 and the threshold *θ *on *r*^2 ^for uncorrelated SNPs was set to 0.1. Please see Methods for an analysis of how performance is affected by variations of the parameters *B *and *θ*.

Independent train-test set pairs for assessing the classification performance of BoNB were obtained by repeatedly sub-sampling at random 90% of the dataset for training and 10% for testing. The procedure was iterated 10 times and classification performance was assessed with the MCC of the predictions on the test sets. The list of selected biomarkers, on the other hand, was computed on the whole dataset.

Classification performance was compared with the ones obtained by a standard Naïve Bayes Classifier, trained on all the SNPs that reached the significance threshold of 5 × 10^-7 ^(as in [7]) in a single 2*df χ*^2 ^test of association with a general genetic model, and by HyperLASSO, a logistic regression method for the simultaneous analysis of all SNPs in a genome-wide association study [10]. The former algorithm was chosen to assess the improvement of BoNB both in terms of biomarker selection, with respect to a standard univariate test, and in terms of classification performance, with respect to the algorithm on which BoNB is based. The latter algorithm was chosen because of its best performance among classification and biomarker selection methods for genome-wide data, as reported in [10] and [20], and because of the complete availability of the source code (see Methods for further details on the Naïve Bayes and HyperLASSO algorithms).

**Figure 2:**
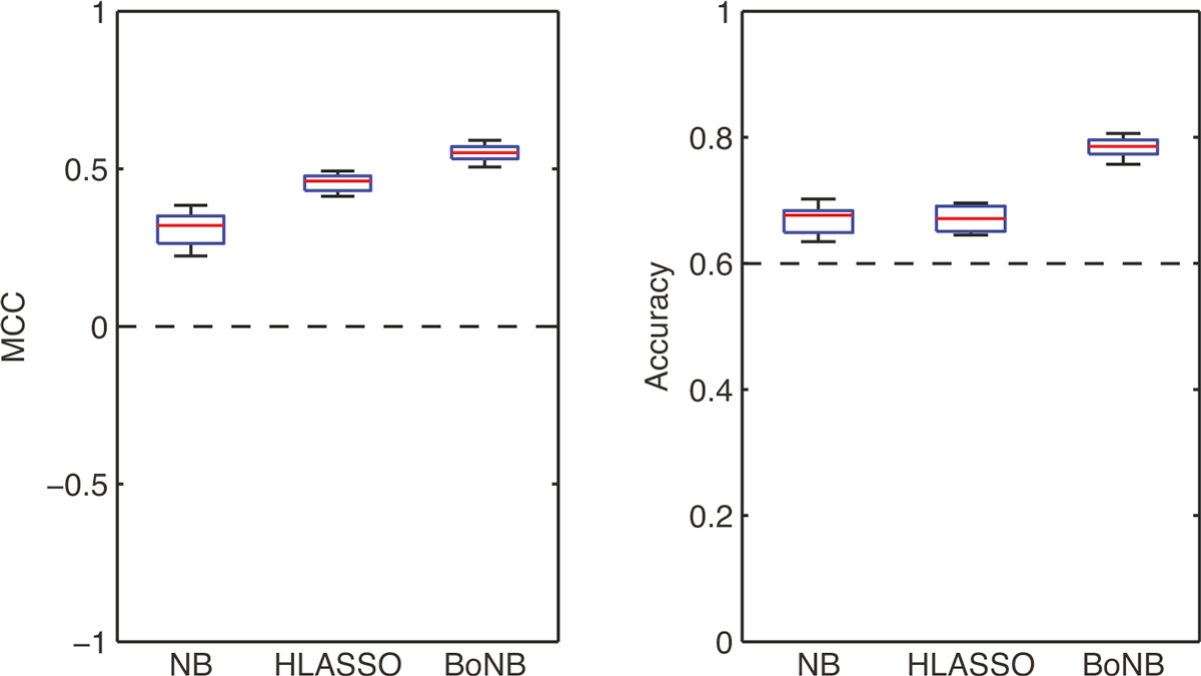
**Box plots of MCC (left panel) and classification accuracy (right panel) of the standard Naïve Bayes classifier, HyperLASSO and BoNB on ten random subsamplings of the WTCCC T1D dataset**. The dashed lines represent the classification performance of a majority classifier.

On the experimental dataset, BoNB reached an MCC of 0.55 ± 0.03 (mean ± standard deviation), significantly higher than the ones reached by both the standard Naïve Bayes Classifier (0.31 ± 0.05, Wilcoxon signed-rank p-value 0.002) and by HyperLASSO (0.46 ± 0.03, p-value 0.002). Figure 2 (left panel) shows the boxplots of the MCC obtained by the three algorithms on the ten iterations of the sub-sampling procedure. For the sake of completeness, Figure 2 (right panel) shows also the boxplots of classification accuracy. The dashed lines in the two plots represent the classification performance of a majority classifier.

**Figure 3:**
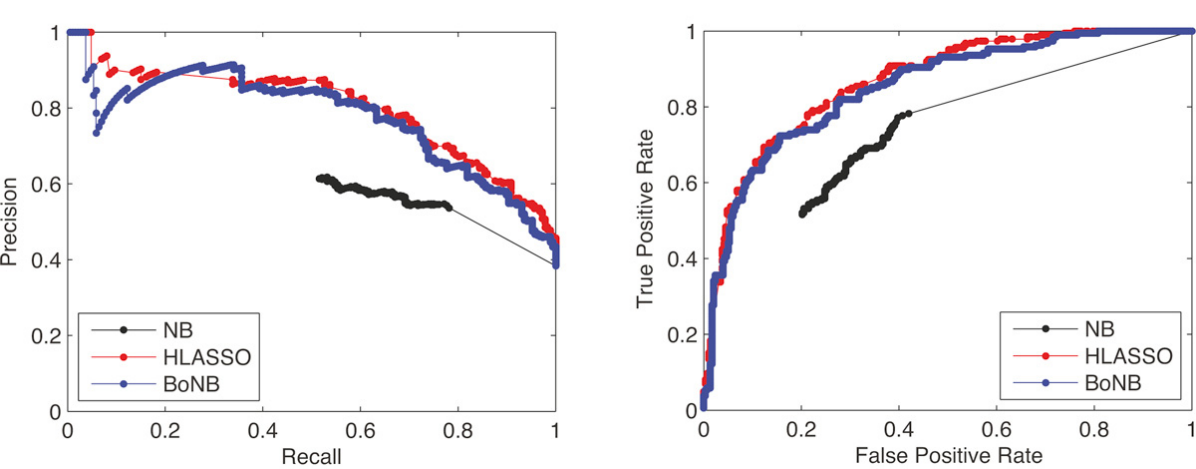
**Precision *vs *Recall curve (left panel) and Receiver Operating Characteristic (right panel) of the standard Naïve Bayes classifier, HyperLASSO and BoNB on a random subsampling of the WTCCC T1D dataset**.

To further analyze the behaviour of the three methods at different levels of the output function (*i.e*. of the output class probability for BoNB and the standard Naïve Bayes classifier and of the logistic regression value for HyperLASSO) we report in Figure 3 the Precision *vs *Recall curve and the Receiver Operating Characteristic, or True Positive Rate *vs *True Negative Rate curve, of the three algorithms on one of the ten random subsamplings (the behaviour on the other subsamplings is similar). As it is clear from the figure, the performance of the standard Naïve Bayes classifier is completely dominated by the performance of both BoNB and HyperLASSO. Concerning the two latter algorithms, one can observe that HyperLASSO has a better performance at the two extremities of the curves, *i.e*. for subjects whose logistic regression value is closer to the maximum or the minimum; moving from the extremities to the middle scores, BoNB outperforms HyperLASSO, being indeed able to reach overall higher MCC and classification accuracy.

For biomarker selection, we run BoNB on the whole dataset and compared its results with the biomarkers identified by HyperLASSO and by the general 2*df *test. The average number of attributes included by BoNB in each NBC was 3.24, 75 SNPs were included by at least one NBC and 9 SNPs by at least 5% of the NBCs (see Table 1). Among the 9 SNPs, only 7 SNPs reached the significance level on the permutation test and were chosen as genetic biomarkers (marked in bold in Table 1). All the 7 selected SNPs fall into regions of interest for Type 1 Diabetes according to the on-line database T1DBase [21] (cytobands p13.2 on chromosome 1 and p21.32 on chromosome 6, also known as the MHC region) and their association with the disease was confirmed in a larger meta-analysis, subsequent to the WTCCC study [9]. The squared correlation coefficients between all pairs of selected SNPs are all lower than 0.155, indicating low redundancy in the information coded by the set of 7 SNPs.

**Table 1:** SNPs selected as attributes for at least 5% of the Naïve Bayes Classifiers by BoNB on the WTCCC T1D dataset, with *B *= 200 Bootstrap samples and classifiers.

SNP	Chr	Gene	Relation	%NBCs	MU (median)
**rs6679677**	**1**	**RSBN1**	**downstream**	**7**	**0.033**
rs9266774	6	MICA	upstream	5.5	0.011
**rs805301**	**6**	**BAT3**	**intron**	**17.5**	**0.043**
**rs492899**	**6**	**SKIV2L**	**intron**	**8.5**	**0.025**
**rs9273363**	**6**	**HLA-DQB1**	**downstream**	**100**	**0.835**
**rs9275418**	**6**	**HLA-DQB1**	**upstream**	**80**	**0.160**
**rs6936863**	**6**	**HLA-DQA2**	**upstream**	**8**	**0.08**
rs9784858	6	TAP2	intron	5	0.008
**rs3101942**	**6**	**LOC100294145**	**exon**	**21.5**	**0.045**

First column: dbSNP RS ID. Second column: SNP chromosome. Third and fourth column: annotated gene and relation with the SNP. Fifth column: percentage of Naïve Bayes Classifiers that included the SNP as attribute. Sixth column: median of the marginal utility of the SNP. SNPs selected as genetic biomarkers by the permutation procedure are marked in bold.

Compared to the 394 SNPs that reached the significance level on the 2*df *general test, both the list of 75 SNPs used for classification and the list of 7 biomarkers selected by BoNB are more compact, but this does not prevent BoNB to reach significantly higher classification performance.

HyperLASSO selected 8 SNPs, all in the MHC region of chromosome 6: 4 of the SNPs are among the biomarkers selected by BoNB, thus suggesting a certain coherence between the two algorithms and providing further confidence on the identified biomarkers.

### Implementation

BoNB is implemented in C++ and relies only on standard libraries, thus being fully portable across operating systems. On the WTCCC case-control study on Type 1 Diabetes, BoNB takes approximately 50 minutes for training 200 NBCs and selecting the biomarkers on a 3.00 GHz Intel Xeon Processor E5450. A careful allocation strategy makes BoNB occupy around 600 MB of RAM for the WTCCC dataset, allowing it to be easily run on a desktop computer.

## Discussion

In this paper, we presented a novel algorithm for classification and biomarker selection from genome-wide SNP data. The algorithm, Bag of Naïve Bayes (BoNB), is based on the Naïve Bayes classification framework, enriched by three main features: bootstrap aggregating of an ensemble of Naïve Bayes classifiers, a novel strategy for ranking and selecting the attributes used by each classifier and a permutation-based procedure for selecting significant biomarkers, based on their marginal utility in the classification process.

The effectiveness of BoNB was demonstrated by applying it to the WTCCC case-control study on Type 1 Diabetes: BoNB indeed outperforms two algorithms from the state of the art, namely a Naïve Bayes Classifier and HyperLASSO, in terms of classification performance and all the genetic biomarkers identified by BoNB are meaningful for Type 1 Diabetes.

Learning an ensemble of classifiers from a bootstrap sample of the original dataset provides BoNB with two main advantages: on the one hand, it guarantees a higher generalization ability by increasing the stability of the learning process [17]; on the other hand, it allows to define a measure of the marginal utility of each SNP, given all the other SNPs exploited for classification, and to select significant biomarkers among these SNPs in a sound and statistically principled way.

Two features of the Naïve Bayes Classifier, chosen as building block of the BoNB algorithm, make it rather appealing for genome-wide data analysis: on the one hand, conditional probability table analysis does not assume a pre-specified model of genetic effect, on the other hand, missing values are seamlessly handled by both the learning and the classification procedure.

The idea of bagging Naïve Bayes classifiers has already been proposed in the Random Naïve Bayes algorithm of Prinzie and Van der Poel [22]. The authors suggest, as a means for enforcing independence between the attributes of each NBC, to sample the attributes at random from the whole attribute set. Such an approach, however, is unfit to genome-wide data analysis: the number of informative attributes is largely lower than the total number of attributes and the probability of capturing them by random sampling is thus extremely low.

Our approach to attribute selection, consisting in a univariate ranking step followed by a multivariate selection step, has the advantage of favouring informative attributes, but without the need of pre-selecting fixed sets of attributes or of defining cut-offs on the strength of the association with the disease: attributes, in fact, are added to the classifiers as long as their combined effect on the generalization ability increases.

**Figure 4:**
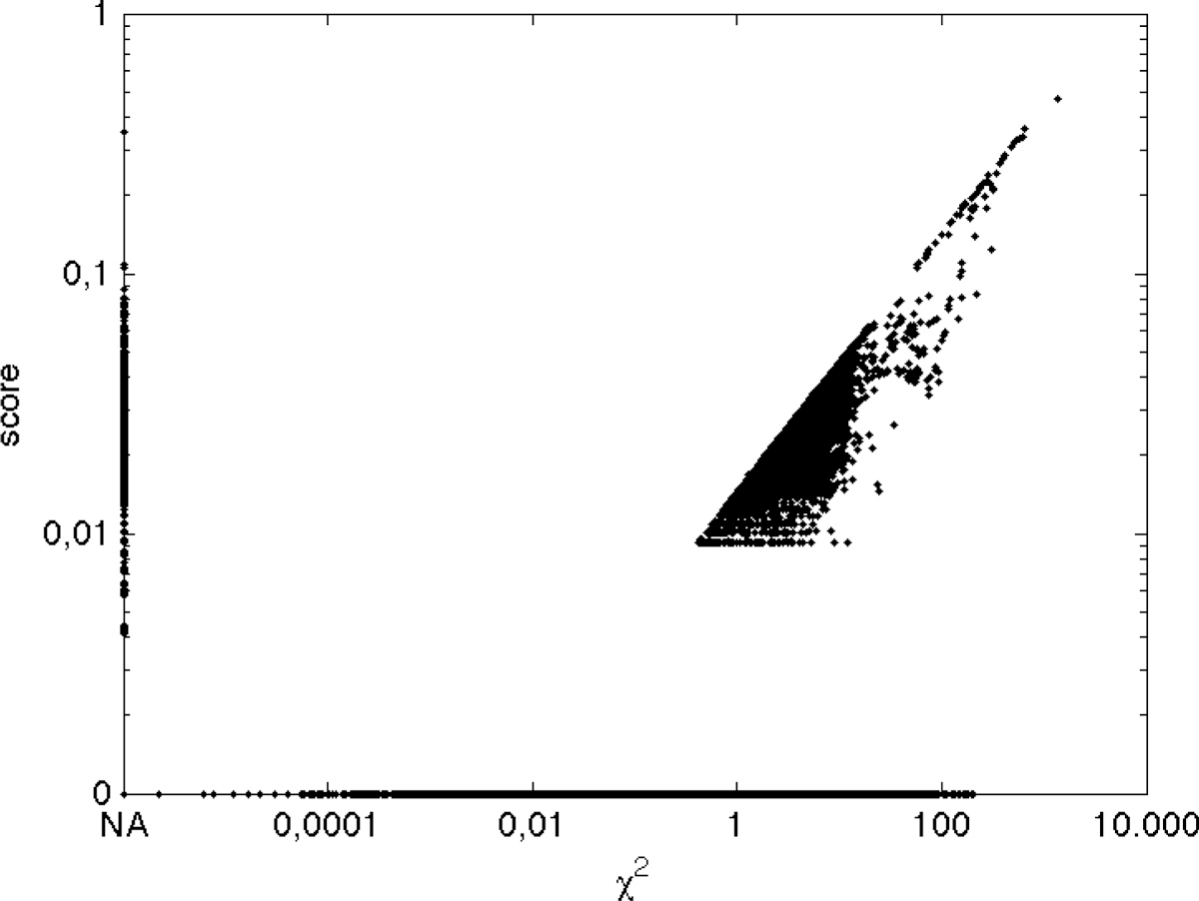
**Naïve Bayes attribute score *vs χ*^2 ^statistic for all SNPs in the WTCCC T1D dataset**.

To provide the reader with further insight on the Naïve Bayes attribute score, exploited in BoNB for univariate attribute ranking, we studied it against the 2df *χ*^2 ^statistic of association for all the SNPs in the Wellcome Trust Case-Control Study on Type 1 Diabetes (Figure 4).

As it can be seen from the figure, the two measures are in a strong monotonic relation for the majority of SNPs; when used as ranking criteria, thus, they are deemed to return similar ranked lists.

Major exceptions are the points plotted along the two axes of Figure 4. Along the vertical axis lie the SNPs for which the *χ*^2 ^test can not be run, because at least one of the entries in the SNP contingency table has less than 5 elements. Along the horizontal axis, on the other hand, lie the SNPs that, when used to train a Naïve Bayes Classifier, lead to a majority classifier, *i.e*. a classifier that always returns the most frequent of the two classes as output: this happens when one of the classes is the most represented for all the three genotypes of a SNP.

Analyzing the extreme behaviours of the two scoring measures provides the key for understanding the main difference between them: while *χ*^2 ^is designed to capture a difference in SNP frequencies from the frequencies expected under no association between the SNP and the disease, the Naïve Bayes attribute score is meant to select good *predictors *of the disease, under the Naïve Bayes classification model.

For this reason, the Naïve Bayes attribute score is not much sensitive to small variations of contingency table entries with few or zero elements and thus it does not require a minimum number of elements per entry. On the other hand, it does not reward SNPs with even large differences in frequencies from the case of no association, if one of the two classes is consistently over represented, since such SNPs can not be effective as univariate predictors in the dataset under analysis.

## Conclusion

The analysis of genome-wide SNP data for multifactorial diseases mainly suffers from two, intertwined problems: on the one hand, multifactorial diseases are caused by complex patterns of interaction between multiple genetic traits and the environment, on the other hand, genetic linkage confounds the search for genetic biomarkers, because of the non-random association between the true genetic causes and the SNPs in genomic regions close to them. The algorithm we proposed, Bag of Naïve Bayes, proved effective in tackling both of these problems: the simultaneous analysis of all SNPs on a genome-wide scale can capture the sets of SNPs with the strongest joint effect on the disease; the novel procedure for attribute ranking and selection enforces attributes independence, thus discriminating causal SNPs from nearby weaker signals.

Apart from genome-wide association studies, BoNB can also be applied, with minor modifications, to the analysis of SNP data from case/control exome sequencing experiments [23,24]: in this scenario, given the lower average distance between SNPs on the same genes and the consequently stronger effect of genetic linkage, the ability of BoNB to enforce attribute independence would prove even more useful.

## Methods

### Naïve Bayes Algorithm

The Naïve Bayes Algorithm [15] for discrete-valued inputs and binary classification learns the probability distributions of Equation 1 estimating two sets of parameters. The first is:

(3) $$\theta_{ijk}=\text{Pr}\left(X_{i}=x_{ij}|Y=y_{k}\right)≃\frac{\#D\left\{X_{i}=x_{ij}\wedge Y=y_{k}\right\}+l}{\#D\left\{Y=y_{k}\right\}+lJ},\;j=1\ldots J$$

for each input attribute *X_i_*, each of its possible values *x_ij _*(*J *= 3 in case of SNPs) and each of the two possible values *y_k _*of *Y*. The #*D *{*x*} operator returns the number of elements in the training set *D *that satisfy property *x*.

In addition, the algorithm must estimate the parameters of the *prior *probability over *Y*:

(4) $$\pi_{k}=\text{Pr}\left(Y=y_{k}\right)≃\frac{\#D\left\{Y=y_{k}\right\}+l}{|D|+2l}$$

where |*D*| denotes the number of elements in the training set *D*.

The only tunable parameter of the Naïve Bayes Algorithm is the *l *term, known in the Bayesian literature as Equivalent Sample Size or Dirichlet Weight. Both for the ensemble of classifiers exploited by the BoNB algorithm and for the standard Naïve Bayes algorithm used in the comparison *l *was fixed to 1, implementing what is called a Laplace smoothing [15].

### Naïve Bayes attribute score

In the BoNB algorithm, SNPs are ranked as candidate attributes of the *b*-th Naïve Bayes Classifier according to their ability in discriminating the subjects in the Bootstrap replicate **X**^(*b*)^; this is estimated for each SNP as the MCC of a Naïve Bayes Classifier, trained and tested on **X**^(*b*)^, with the SNP as the single attribute. The rationale for such a measure is to give a higher rank to SNPs that guarantee a lower training error on **X**^(*b*) ^when used as attributes.

For a more formal definition of the score, we start by defining the elements of the contingency table for the SNP as in Table 2. Considering that the parameters of a Naïve Bayes Classifier are estimated according to Equations (3) and (4) (presented in the previous section) and that we set the Dirichlet Weight *l *to 1, we can define the three following inequalities:

$$\begin{array}{c} I_{0}:\;\frac{a+1}{n_{ca}+3}\;\;\cdot \;\;\frac{n_{ca}+1}{n+2}>\frac{d+1}{n_{co}+3}\;\;\cdot \;\;\frac{n_{co}+1}{n+2}\\ I_{1}:\;\frac{b+1}{n_{ca}+3}\;\;\cdot \;\;\frac{n_{ca}+1}{n+2}>\frac{e+1}{n_{co}+3}\;\;\cdot \;\;\frac{n_{co}+1}{n+2}\\ I_{2}:\;\frac{c+1}{n_{ca}+3}\;\cdot \;\;\frac{n_{ca}+1}{n+2}>\frac{f+1}{n_{co}+3}\;\;\cdot \;\;\frac{n_{co}+1}{n+2} \end{array}$$

and the three corresponding indicator functions *I*_0_, *I*_1 _and *I*_2_, returning 1 if the inequality holds and 0 otherwise. The three inequalities determine the behaviour of the Naïve Bayes Classifier in classifying unseen subjects according to their genotype, by comparing the posterior probabilities of the two classes.

When the same set **X**^(*b*) ^is used both for training and for testing, the Naïve Bayes attribute score *S *can be computed as the MCC of the prediction from Equation (2):

(5) $$S=\frac{\left(ae-bd\right)\left(I_{0}-I_{1}\right)+\left(af-cd\right)\left(I_{0}-I_{2}\right)+\left(bf-ce\right)\left(I_{1}-I_{2}\right)}{\sqrt{n_{ca}n_{co}\cdot \left[n_{0}n_{1}\cdot \text{XOR}\left(I_{0},I_{1}\right)+n_{0}n_{2}\cdot \text{XOR}\left(I_{0},I_{2}\right)+n_{1}n_{2}\cdot \text{XOR}\left(I_{1},I_{2}\right)\right]}},$$

where XOR (·,·) is the boolean operator returning 1 if exactly one of the operands is equal to 1, and 0 otherwise.

**Table 2:** Contingency table of a SNP, with the genotype codes 0 for the homozygous pair of minor alleles, 1 for the heterozygous pair and 2 for the homozygous pair of major alleles.

genotype	0	1	2	
		
cases	*a*	*b*	*c*	*n_ca_*
		
controls	*d*	*e*	*f*	*n_co_*
		
	*n*_0_	*n*_1_	*n*_2_	*n*

Each element in the contingency table reports the number of subjects with the corresponding genotype and phenotype. *n*_0_, *n*_1 _and *n*_2 _are the column sums, *n_ca _*and *n_co _*are the row sums and *n *is the total subject count for the SNP.

### HyperLASSO algorithm

The HyperLASSO algorithm [10] is a Bayesian-inspired penalized logistic regression approach exploiting a NEG prior for the attribute weights. The NEG prior is a continuous prior distribution with a sharp mode at zero, which has the effect of shrinking the regression weights heavily when they are near zero. The NEG prior has a shape parameter, which we set to the default value 0.1.

Like all other penalized logistic regression approaches, the HyperLASSO algorithm has a tunable parameter *λ *controlling the relative weight of the model complexity (*i.e*. of the number of SNPs included in the classifier) versus the model likelihood. We tested several values of *λ*, namely 50, 100, 250 and 500, and chose the one leading to the best classification performance on the WTCCC T1D dataset (*λ *= 250).

The HyperLASSO algorithm has an element of stochasticity, namely in the order with which model parameters are updated in the model selection procedure, and is designed to carry out multiple runs with different orderings and report the best scoring model. For our analysis, we set the number of runs to 10, resulting in approximately 60 hours for processing the entire WTCCC T1D dataset on a 3.00 GHz Intel Xeon Processor E5450.

### Effect of parameters variation on the performance of BoNB

The BoNB algorithm exposes two parameters to the user: the number of Bootstrap replicates and Naïve Bayes Classifiers, *B*, and the threshold on the squared correlation coefficient above which two SNPs are considered correlated, *θ*.

**Figure 5:**
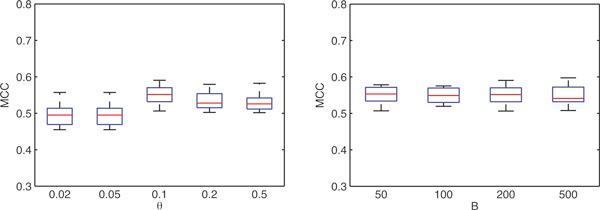
**Box plots of the MCC obtained by BoNB on ten random subsamplings of the WTCCC T1D dataset, for *B *= 200 and *θ *ranging from 0.02 to 0.5 (left panel) and for *θ *= 0.1 and *B *ranging from 50 to 500 (right panel)**.

Figure 5, left panel, represents the MCC obtained by BoNB on ten random subsamplings of the WTCCC T1D dataset, for *B *= 200 and *θ *ranging from 0.02 to 0.5. As it is clear from the figure, *θ *= 0.1 is optimal and results in a significantly higher classification performance (Kruskal-Wallis test p-value 3.7 × 10^-4^).

Concerning the number of Bootstrap replicates *B*, on the other hand, one can observe from Figure 5, right panel, that classification performance is not much sensitive to variations of *B *(Kruskal-Wallis test p-value 0.98), though it is slightly higher for *B *= 50 and 200. Analyzing the list of selected biomarkers, BoNB returns the same seven biomarkers reported in Table 1 for *B *= 200 and 500, adds SNP rs2856688 to the list for *B *= 100 and misses SNPs rs6679677 and rs492899 for *B *= 50.

Given the consistency among the results for higher values of *B*, suggested values for BoNB parameters are thus *θ *= 0.1 and *B *= 200.

## List of abbreviations

SNP: Single Nucleotide Polymorphism; GWAS: Genome-Wide Association Study; NBC: Naïve Bayes Classifier; OOB: Out-of-Bag; MCC: Matthews Correlation Coefficient; MU: Marginal Utility.

## Competing interests

The authors declare that they have no competing interests.

## Authors' contributions

FS designed and implemented the BoNB algorithm, carried out its performance analysis and drafted the manuscript. ET carried out the performance analysis of the algorithms for the comparison. BDC participated in the design of both the algorithm and the performance analysis and helped to draft the manuscript. GMT helped to design the performance analysis and to draft the manuscript. CC coordinated the study and helped to draft the manuscript. All authors read and approved the final manuscript.
